# Newer Insights on the Occurrence of Sarcopenia in Pediatric Patients with Cancer: A Systematic Review of the Past 5 Years of Literature

**DOI:** 10.3390/cancers17193188

**Published:** 2025-09-30

**Authors:** Georgios Kiosis, Despoina Ioannou, Kanellos Skourtsidis, Vasilis Fouskas, Konstantinos Stergiou, Dimitrios Kavvadas, Theodora Papamitsou, Sofia Karachrysafi, Maria Kourti

**Affiliations:** 1 Research Team “Histologistas”, Interinstitutional Postgraduate Program “Health and Environmental Factors”, School of Medicine, Faculty of Health Sciences, Aristotle University of Thessaloniki, 54124 Thessaloniki, Greece; dioana@auth.gr (D.I.); kskour@auth.gr (K.S.); fvasillis79@gmail.com (V.F.); konstantinos.d.stergiou@outlook.com (K.S.); kavvadas@auth.gr (D.K.); thpapami@auth.gr (T.P.); sofia_karachrysafi@outlook.com (S.K.); 2 Laboratory of Histology-Embryology, School of Medicine, Faculty of Health Sciences, Aristotle University of Thessaloniki, 54124 Thessaloniki, Greece; 3 Department of Radiology, General Hospital AHEPA, 54124 Thessaloniki, Greece; 4 Pediatric & Adolescent Hematology Oncology Unit, 2nd Pediatric Department, Faculty of Health Sciences, Aristotle University of Thessaloniki, AHEPA Hospital, 54124 Thessaloniki, Greece

**Keywords:** sarcopenia, muscle mass loss, muscle loss, muscle weakness, neoplasm, cancer, malignac*, oncology, child, pediatric

## Abstract

**Simple Summary:**

Children with cancer often face many challenges not only from the disease itself but also from the side effects of treatment. One of the most serious problems is the loss of muscle mass and strength, a condition called sarcopenia. This condition makes recovery harder, reduces the body’s ability to heal infections, and can even affect the success of cancer treatment. In this review, we looked at studies from the last five years that examined sarcopenia in children with different types of cancer. We found that sarcopenia is common both during and after treatment. It can appear before surgery or chemotherapy and may continue long after treatment has ended, leading to long-term health problems such as reduced bone strength, frailty, and higher risk of relapse. Although methods to measure sarcopenia in children are not yet standardized, approaches such as imaging scans and body composition tests are being used more often. Early detection is crucial, because simple steps like proper nutrition and physical activity programs can help children maintain or rebuild muscle. Understanding and addressing sarcopenia is vital to improve survival, reduce complications, and ensure better quality of life for young cancer patients and survivors.

**Abstract:**

Background/Objectives: Sarcopenia, defined as the progressive loss of muscle mass and function, is increasingly recognized in pediatric cancer patients as a significant clinical and prognostic factor. Sarcopenia in children arises from malignancy-related inflammation, malnutrition, and treatment toxicity, negatively affecting treatment response, recovery, and quality of life. Methods: We searched MEDLINE and Scopus for English-written articles published over the last five years using synonyms for the terms “sarcopenia” and “pediatric cancer”. Screening and data extraction were performed in a duplicate-blinded method. We qualitatively synthesized eligible articles. Results: Recent studies identify pre-treatment sarcopenia as a marker of poor prognosis, especially in hepatoblastoma and neuroblastoma. Total psoas muscle area (tax) and skeletal muscle index (SMI) are emerging diagnostic tools, though standardized methods remain lacking. Sarcopenia’s etiology is multifactorial, involving impaired mitochondrial metabolism, chemotherapy-induced appetite loss, and systemic inflammation. Sarcopenic obesity is common, particularly among leukemia survivors, often masked by normal BMI. Survivors also face reduced bone density, impaired immunity, and persistent muscle loss, linked to prior therapies such as radiotherapy and hematopoietic stem cell transplantation. Increase in muscle mass post-treatment correlates with better survival outcomes. Conclusions: Early detection of sarcopenia can support timely interventions such as nutritional support and physical activity. Yet, significant diagnostic heterogeneity across existing studies hampers definitive conclusions regarding its true prevalence and the optimal assessment method. Standardized diagnostic criteria are urgently needed to enable more reliable prevalence estimates and evidence-based clinical strategies.

## 1. Introduction

### 1.1. Pediatric Cancer

The term pediatric cancer refers to a complex and multifaceted disease, with various causes and significant consequences for both the affected child and their family as a whole. Genetic and epigenetic changes [1,2], structural and numerical chromosomal abnormalities [3], ionizing radiation, immunosuppression, infection with oncogenic viruses, as well as prior cancer treatments, are among the recognized causes of childhood cancer development [4]. In addition, the International Agency for Research on Cancer (IARC) categorizes ionizing radiation as a Group 1 carcinogen, meaning it is known to cause cancer in humans [5]. Birth defects, in addition, increase childhood cancer risk, especially with multiple defects [6]. The strongest link appears in Down syndrome that raises the risk of acute lymphoblastic leukemia (ALL) 20-fold and acute megakaryocytic leukemia (AML) 500-fold [7,8]. Klinefelter syndrome is linked to germ cell tumors, as well [9]. Nervous system defects are associated with brain tumors [10], and other defects with neuroblastoma and hepatoblastoma [6,8]. Sunburn and sunlight exposure during childhood increase the risk of melanoma and other skin cancers diagnosed later in life [11].

Pediatric cancers are typically of mesodermal origin and represent the most common disease-related cause of death among children. Pediatric tumors generally exhibit a lower mutational burden, typically driven by a singular clonal genetic event such as a translocation resulting in an oncogenic fusion [12]. These tumors characteristically display limited immune cell infiltration and are frequently classified as immunologically “cold” [13,14]. The most frequent types include leukemia (24.7%), central nervous system tumors (17.2%), non-Hodgkin lymphoma (7.5%), Hodgkin lymphoma (6.5%), and soft tissue sarcomas (5.9%) [15]. Due to their heterogeneity, the World Health Organization’s classification of pediatric tumors has emphasized molecular and genetic characteristics, enabling more precise diagnosis and treatment [15].

Significant progress has been made in the treatment of pediatric cancer over the past few decades. The introduction of innovative approaches, such as immunotherapy [16,17,18] and targeted treatments, has contributed to the improvement of survival outcomes and the reduction in side effects. Furthermore, care for pediatric cancer patients must extend beyond the end of therapy [19].

The steadily growing population of childhood cancer survivors frequently experiences physical and psychological complications, which may stem from either the malignancy itself or its treatment [20]. Chemotherapy, the standard treatment option for many pediatric cancers [21,22], often causes considerable side effects [23,24], highlighting the urgent need for less toxic alternatives [25]. In addition to drug toxicity, multi-drug resistance (MDR) remains a major challenge in pediatric cancer treatment, including chemotherapy, targeted therapy, and immunotherapy [26,27]. Cancer cells may acquire resistance to a specific chemotherapeutic agent and concurrently to others with distinct chemical structures and mechanisms, a phenomenon referred to as MDR [28,29,30].

### 1.2. Correlation of Sarcopenia with Pediatric Cancer

Sarcopenia is defined as the progressive and generalized loss of muscle mass and function [31]. Although traditionally recognized as a pathological condition more commonly present in the elderly, increasing data from various studies show that sarcopenia can also occur in younger individuals, particularly in children suffering from cancer, either hematologic or solid. These patients experience muscle mass loss due to the combined adverse effects of malignancy, inflammation, malnutrition, and the toxicity of therapeutic options, such as chemotherapy and radiotherapy [31,32,33,34]. In pediatric oncology patients, sarcopenia is consistently associated with treatment resistance, increased incidence of infections, and worse surgical prognosis, especially when the child undergoes surgical tumor removal [35,36,37].

Sarcopenia is not a condition that occurs only during treatment, as newer data confirm that it can also be present after the end of the therapy, affecting long-term survivors of childhood cancer. This condition significantly burdens their physical health and functionality as well as their overall quality of life [32,38,39]. Newer research data confirm the presence of sarcopenia either during or after treatment, using imaging methods such as DXA, CT, and MRI. These methods contribute to the understanding of changes in muscle mass; however, the heterogeneity that characterizes them poses a significant limitation in the comparability of results and the broader application of their findings [40,41]. In certain types of cancer, such as childhood leukemia and lymphoma, and specifically in survivors of these diseases, sarcopenia has been found to coexist with increased fat mass, a condition known as sarcopenic obesity. This creates additional problems for patients and further complicates the clinical picture [42,43,44].

Sarcopenia appears to affect not only the patients’ functionality but also their bone health and skeletal development. Thus, there is an urgent need for multifactorial monitoring of these patients as well as the application of specialized treatment [35,41]. The systematic evaluation of the musculoskeletal system serves as an early indicator for complications and guides treatment adjustments, as several studies confirm [45].

Since sarcopenia is an established pathological condition in pediatric oncology patients, more and more studies today are focusing their attention on calculating its prevalence, as well as identifying diagnostic criteria for its recognition and subsequent management. However, despite these efforts, to date, there is no universally accepted standardized diagnostic approach for sarcopenia in children. This creates confusion and leads to inconsistencies in its diagnosis [46]. Apart from the reviews that aim to understand the landscape of sarcopenia in children, several studies are investigating potential interventions to address its consequences. These interventions primarily involve the implementation of appropriate nutrition and physical exercise. Initial results from these methods are encouraging [47,48].

Given the clinical and prognostic significance of sarcopenia in children with cancer and the continuously evolving literature, a comprehensive synthesis of the latest data is deemed necessary. This systematic review aims to provide an overall overview of sarcopenia in children with cancer, based on data from the last 5 years, highlighting trends in prevalence, diagnostic approaches, and clinical outcomes.

## 2. Materials and Methods

Our systematic review was designed and reported in compliance with the Preferred Reporting Items for Systematic Reviews and Meta-Analyses (PRISMA) guidelines. A completed PRISMA checklist has been compiled and is available in the Supplementary Material (S1). Regarding our search strategy and eligibility criteria, we performed a search in the electronic databases PubMed/MEDLINE and Scopus covering all the papers from 2020 until 26 May 2025. The search strategy employed the following combination of keywords: (“sarcopenia” OR “muscle mass loss” OR “muscle loss” OR “muscle weakness”) AND (“neoplasm*” OR “cancer*” OR “malignanc*” OR “oncology”) AND (“child*” OR “pediatric”).

We included studies in children and adolescents with coexistence of pediatric cancer, either during or after treatment, and sarcopenia. Eligible publication types included original research articles, systematic reviews, meta-analyses, and case reports directly related to the research objective. We only included peer-reviewed articles, with their full text available in English.

Exclusion criteria: studies not meeting the PICO framework (Table 1), non-English publications, or studies not involving human participants. No automation tools were used in any stage of the selection.

**Figure 1:**
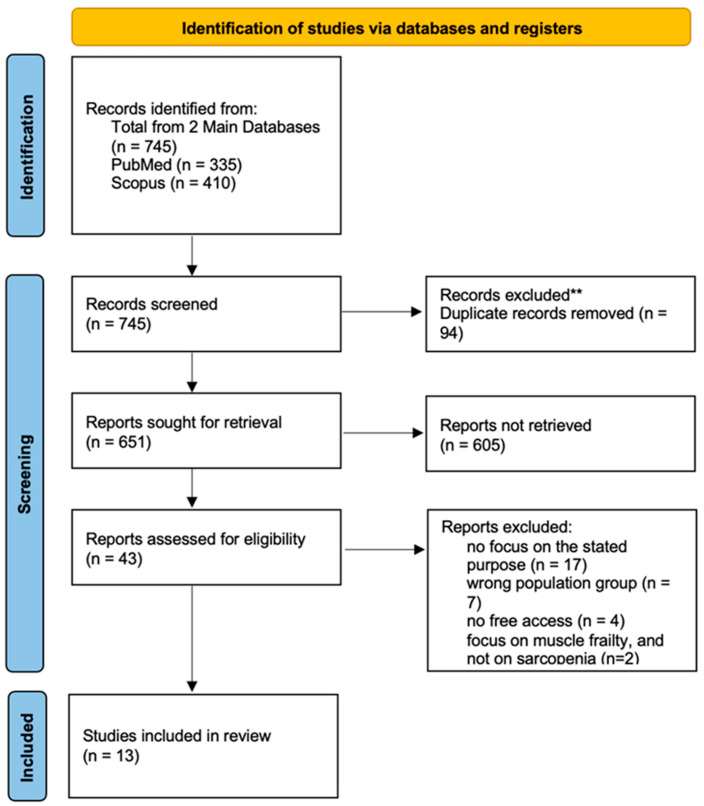
PRISMA Flow Diagram.

Regarding our data management, potentially eligible articles were exported into “Rayyan” (2016), a web-based application for article screening. Via Rayyan duplicates were removed. Two reviewers independently screened titles and abstracts and subsequently full texts, remaining blinded to each other’s decisions during the process. Conflicts were resolved with the involvement of a third investigator. The PRISMA flow diagram was constructed and presents the progress from initial studies to final results. (Figure 1).

Data extraction was performed in duplicate by two independent investigators, based on predetermined forms, with the involvement of a third reviewer in case of discrepancies. Key data extracted included author, publication date, study design, cancer type, study population, main findings related to sarcopenia, and assessment methods (e.g., tPMA, SMI, BIA). Studies were synthesized narratively due to methodological heterogeneity across populations, interventions, and outcomes. A summarizing table of all the studies is mentioned above. (Table 2) Two independent reviewers assessed the methodological quality. The methodological quality of included cohort studies was assessed using the Newcastle–Ottawa Scale (NOS). This tool evaluates study quality based on three domains: selection, comparability, and outcome. Each study was rated out of a maximum of 8 points. Quality assessment results are presented in Table 3.

Several included studies were rated as having moderate methodological quality (NOS 5–6), and their limitations should be considered when interpreting the overall conclusions. Romano et al. (2024) [50] scored 5 points, mainly due to a very small sample size (14 participants) and lack of adjustment for potential confounders, which reduces the external validity of its findings on sarcopenia and metabolic syndrome in brain tumor survivors. Buğdaycı et al. (2023) [59] (score 6) was limited by incomplete adjustment for additional risk factors and a relatively modest cohort of 60 patients, which may have reduced the power to detect prognostic associations between sarcopenia and survival in sarcoma. Omori et al. (2022) [60] (score 6) did not adjust for other risk factors and had limited representativeness due to its small progression-free/relapse groups, which raises concerns about selection bias. Collectively, while these studies support the association between sarcopenia and adverse outcomes, their methodological constraints mean their findings should be interpreted with caution. Nevertheless, their consistency with higher-quality studies strengthens the overall conclusion that sarcopenia is a clinically significant concern in pediatric oncology.

## 3. Results

### 3.1. PRISMA Flow Diagram

Initially, the total number of articles found without applying the criteria was recorded. The total number of articles found was 745, 335 being from PubMed and 410 from Scopus. After the first screening applied; a 5-year publication window, review articles (literature, systematic and meta-analyses), cohort studies, research articles, and case reports, open access and published in the English, language—699 articles were excluded as ineligible. Thus, 221 articles were recorded and screened again. Moving on, 46 final articles were assessed for eligibility, from which a total of 30 articles were excluded for the following reasons—27 articles were excluded because they were not focused on the stated purpose, 13 due to wrong study population, 4 due to inaccessible reports or not available full text, and 2 due to focus on muscle frailty and not on sarcopenia.

A table [Table 2] was then prepared to summarize the findings of those eight articles with the following columns: study, type of study, association sarcopenia-cognitive impairment, association frailty-cognitive impairment, and preventing/healing measures.

### 3.2. Main Results

Ritz studied the development of sarcopenia in children with hepatoblastoma, and those with preoperative sarcopenia were classified as high-risk. The total psoas muscle area (tPMA), used for levels L3 and L4–5, is a reliable indicator for diagnosis in children, with established reference values for these specific levels. It is emphasized that there is no clear diagnostic method. The tPMA value was lower in girls than in boys, possibly due to different pharmacokinetics of anticancer drugs between them. The development of sarcopenia in pediatric tumors is attributed to the following: (a) impaired mitochondrial muscle metabolism due to limited physical activity [49], since exercise—both aerobic and anaerobic—improves mitochondrial function, as noted in Song’s study [34]; (b) mutations in the PI3K/AKT/mTOR signaling pathway affect growth; (c) chemotherapy reduces appetite and caloric intake, exacerbating malnutrition; (d) expression of NfKb in muscles due to cisplatin, which causes muscle mass loss; (e) tumor sarcopenia caused by overactivation of STAT3, which leads to muscle mass loss [49]. Kang’s study highlights the preventive administration of adalimumab to reduce sarcopenia [61].

According to Romano [50], treatment complications for pediatric brain tumors include overweight, fat accumulation, and obesity, increasing the risk of metabolic syndrome (MetS), cardiovascular disease, and death [50,62,63]. The study used bioelectrical impedance analysis (BIA), a non-invasive method whose results require further validation with dual-energy X-ray absorptiometry and subcutaneous fat ultrasound. It showed lower values compared to healthy individuals for: basal metabolic rate (BMR), body cell mass (BCM), body cell mass index (BCMI), fat-free mass (FFM), skeletal muscle mass (SM), skeletal muscle mass index (SMI), and appendicular skeletal muscle mass (ASMM) [50]. BCM is the best predictor of malnutrition [64,65,66]. For children with hepatoblastoma, Romano agrees with Ritz [49] regarding low muscle mass.

Das’s study included survivors of pediatric acute lymphoblastic leukemia in India and found that 34% of them had sarcopenic obesity—a combination of muscle mass loss and fat tissue increase. These patients had a normal body mass index (BMI), hence further evaluation is required to avoid underestimating the problem. Age of diagnosis, central obesity, and insulin resistance cause disproportionate fat tissue gain compared to muscle. Younger age of diagnosis and start of treatment were associated with long-term adverse outcomes. Additionally, 20% of survivors had reduced bone density, which was not associated with sarcopenic obesity. The low dose of cranial radiotherapy (CRT) (12.5 Gy or 18 Gy) received by the patients and the use of steroids contributed to sarcopenic obesity [44].

In Kudo’s study, 138 CT images from 36 patients with neuroblastoma (NB) were analyzed. Among the four parameters studied—height (HT), weight (BW), body mass index (BMI), and skeletal muscle index (SMI)—only the SMI measured at diagnosis was associated with overall survival [52]. Sarcopenia that developed post-treatment, especially within the first year of diagnosis, impaired antitumor immunity by preventing immune cells from entering the tumor, thus worsening outcomes [51,67,68]. In addition to SMI, serum albumin was another prognostic factor [51,69,70], correlating with nutrition and chronic inflammation in patients [51,71,72]. Malnutrition and systemic inflammation were associated with worsening of NB [51].

According to Marmol-Perez, 37.9% of pediatric cancer survivors developed sarcopenia, with most of them being male. Survivors with sarcopenia had lower bone mineral density (BMD), and values of male patients were lower than those of females, except at the femoral neck [52]. Marmol-Perez further confirmed the association between low BMD and sarcopenia in another study [53,73]. Since the EWGSOP2 definition of sarcopenia includes muscle strength [74], grip strength was also assessed in the study and showed that patients with low ALMI (appendicular lean muscle mass index), i.e., sarcopenia, had normal grip strength [52].

Suzuki studied young patients with hematologic cancer who underwent chemotherapy before hematopoietic stem cell transplantation. Between hospital admission and transplantation, muscle mass loss was observed in 41.5% of patients, assessed via CT-measured psoas muscle area (PMA). These patients, with sarcopenia, had worse 5-year overall survival compared to those with normal muscle mass. Another finding was that older patients were more likely to develop sarcopenia, due to reduced physical activity and poorer dietary habits [53].

According to Romano, in children with soft tissue and bone sarcomas, malnutrition is a common, multifactorial complication exacerbated by disease, treatment, and social conditions. The body’s inflammatory response leads to increased energy demands and muscle catabolism, while symptoms like nausea and anorexia reduce nutrient intake. Although BMI is widely used, it does not reflect muscle mass, which is significantly reduced in children with cancer [54]. Inflammatory cytokines, oxidative stress, and low protein intake cause secondary sarcopenia [54,75]. During treatment, reduced nutritional intake further exacerbates muscle loss, as evidenced by reduced tPMA in most patients—with only 23% remaining free of sarcopenia after 12 months of therapy [54,76,77].

Ritz, in his August 2021 study, included pediatric patients with neuroblastoma and showed that the majority had low preoperative tPMA. These patients had poorer five-year postoperative survival than those who were not sarcopenic at baseline. Risk factors also include age at diagnosis, tumor histology, and chemotherapy protocol [55,78]. Chemotherapeutic agents are directly linked to sarcopenia and malnutrition, although this study did not show a significant difference in preoperative sarcopenia development between children who received and those who did not receive chemotherapy under the NB2004 protocol. Ritz proposes additional potential factors for sarcopenia development in oncology patients, which include: (1) lack of physical activity, leading to decreased protein synthesis via mTOR and MAPK pathways and increased catabolism via TNF-α and NF-kB [55,79]; (2) endocrine changes, such as increased stress hormone secretion [55,80], ghrelin resistance (a fat tissue hormone) [55,81,82], and vitamin D deficiency [55,83,84]; (3) tumor consumption of glucose and proteins for its metabolism [55,85]; (4) systemic inflammation, indicated by elevated inflammatory markers such as IL-6 [55,86].

Guo reports that high-risk neuroblastoma (HR-NBL) survivors, despite poor growth, do not present significant deficits in bone density, possibly due to the protective effects of autologous stem cell transplantation (ASCT) and fat mass preservation. However, severe and long-lasting sarcopenia problems are observed, with reduced muscle mass and strength even years after therapy completion. This sarcopenia is linked to increased mortality and likely results from multiple factors such as intensive therapy, hormonal and nutritional disorders, and direct treatment effects on growth plates. One such therapy is cis-RA, a vitamin A derivative, which causes osteoporosis and epiphyseal plate fusion. No significant differences in fat mass are observed compared to healthy individuals, possibly due to differences in mesenchymal cell differentiation. These findings underscore the importance of early interventions aimed at muscle strengthening and the prevention of sarcopenia in high-risk neuroblastoma (HR-NBL) survivors [56]. Additionally, patients who underwent ASCT show increased mortality in the first to fifth years post-treatment [56,87].

McCastlain’s study is the first to associate mitochondrial DNA (mtDNA) copy number (circular double-stranded DNA molecule) with sarcopenia development in childhood cancer survivors. Sarcopenia’s prevalence in the survivor sample was 27%, with gender differences, and was more frequent in survivors of central nervous system tumors. Along with low mtDNA copy number, factors positively associated with sarcopenia included female gender, age of diagnosis, time since diagnosis, cancer type, physical inactivity, cranial radiation therapy, and presence of the T allele at chromosome rs9991501 [57].

In the study by Van Atteveld, which aims to evaluate the frequency of pre-frailty, frailty, and sarcopenia in survivors of childhood cancer, the prevalence of pre-frailty, frailty, and sarcopenia among them was found to be 20.3%, 7.4%, and 4.4%, respectively. It is particularly noteworthy that these adverse outcomes emerged during the third decade of the survivors’ lives. Multiple risk factors contributed to these pathological conditions, including radiation (cranial or total body), chemotherapy (particularly high doses of cisplatin), endocrine disorders (such as growth hormone deficiency, hypogonadism, and hyperthyroidism), nutritional deficiencies (vitamin B12 and folic acid), and body mass index (BMI). Early intervention in childhood cancer is necessary to prevent the appearance of pre-frailty, frailty, and sarcopenia, which generally affect the quality of life of these individuals [58].

Buğdaycı, in her study of pediatric patients with Ewing sarcoma and osteosarcoma, evaluated the prognostic significance of sarcopenia and sarcopenic obesity using skeletal muscle mass indices (SMIs) based on computed tomography. Although no significant associations were found between SMI indicators or the sarcopenic obesity index and event-free survival (EFS) or overall survival (OS) in the total sample, subgroup analyses led to the following results. In patients without metastasis at diagnosis, higher values of paraspinal SMI and SMI T12–psoas were associated with longer event-free survival, and no mortality was observed in this subgroup. In metastatic patients, increased values of SMI_T12–paraspinal muscles were significantly associated with improved EFS and OS. Furthermore, male patients with SMI T12–paraspinal muscle values above the median had significantly longer overall survival. Thus, specific muscle mass indicators have prognostic value [59].

Omori, in his study on pediatric patients with malignant solid tumors, claims that an increase in skeletal muscle mass after treatment is associated with a better prognosis. Patients with a rate of change in psoas muscle volume (PMV) ≥ 1.20 had significantly longer progression-free survival (PFS) and overall survival (OS) compared to those with a lower rate of change. Gender, treatment duration, tumor type, and albumin levels did not differ between the two groups; however, patients in the group with a higher rate of change had a lower age at diagnosis and a higher Body Mass Index (BMI). Thus, the importance of large muscle mass for long-term survival becomes clear from the above [60].

## 4. Discussion

The pre-therapeutic diagnosis of sarcopenia in children diagnosed with cancer is important, as it serves as a prognostic factor for the applied treatment and the adverse effects it may cause in patients. In the study by Wadhwa et al. (2022) [88], conducted on 107 children with lymphoma or rhabdomyosarcoma, skeletal muscle density (SMD) was assessed, and it was observed to be associated with toxic responses to treatment in young oncology patients. The diagnosis of sarcopenia enables timely intervention in the therapeutic regimen, by adjusting both the dosage and the type of treatment, to achieve anticancer effect and reduced toxicity [88]. In fact, in the study by Muñoz-Serrano et al. (2023) [89], sarcopenia is recognized as a prognostic factor not only for the development of postoperative complications but also for overall poor survival. The identification of sarcopenia at the time of cancer diagnosis in children renders patients eligible for specific therapeutic protocols aimed at avoiding adverse outcomes. Muñoz-Serrano also reports that sarcopenia leads to a higher rate of metastases, thereby serving as a prognostic indicator for metastatic progression of the disease [89].

Beyond the prognostic significance of sarcopenia in pediatric cancer patients, its early diagnosis also facilitates timely intervention aimed at maintaining, and eventually increasing, muscle mass. The review by Schab and Skoczen (2024) emphasizes the need for early intervention through nutritional support and regular physical exercise, to prevent sarcopenia and improve clinical outcomes [90]. In the study by Joffe et al. (2019) [91], it is noted that changes in body composition, including muscle mass, appear early during treatment. Therefore, incorporating a method for assessing muscle mass is essential, as it enables personalized interventions that can help prevent the progression of sarcopenia once diagnosed [91]. As previously mentioned in this review, sarcopenia may also develop in cancer survivors, negatively affecting their quality of life. The study by Cao (2022) [92] highlights the importance of physical exercise to improve sarcopenia among survivors, once it is diagnosed. Thus, identifying survivors at higher risk for adverse health outcomes—and screening them for sarcopenia followed by exercise-based interventions—yields beneficial results [92].

In general, and as supported by earlier studies, interventions for the prevention of sarcopenia focus primarily on three pillars: nutrition, physical activity, and a multimodal approach. Adequate intake of protein and energy, along with supplementation of vitamin D and other micronutrients, may contribute to the preservation of muscle mass [93]. Moreover, a randomized controlled trial in pediatric acute lymphoblastic leukemia found that supplementation with long-chain polyunsaturated fatty acids (LCPUFAs) attenuated loss of lean body mass during treatment [94]. Early one-year nutritional programs focusing on diet quality and energy balance have also shown positive effects on weight and BMI outcomes in children receiving therapy, highlighting the value of early integration of dietitians into care teams [95,96].

At the same time, the incorporation of physical exercise programs, even during treatment, has been shown to improve muscle strength and physical function. Clinical trials in pediatric oncology demonstrate that structured exercise programs—even when administered in hospital settings during aggressive therapy—can improve muscle strength. For instance, a randomized controlled in-hospital exercise program combining aerobic and strength training in children with solid tumors undergoing chemotherapy led to significant improvements in upper and lower body strength compared to controls [97]. Also, supervised exercise interventions in childhood cancer survivors (CCS), both during and post-treatment, have shown improvements in muscle strength, cardiorespiratory fitness, functional mobility, and quality of life [98,99,100].

Finally, multimodal interventions that combine nutrition and exercise have demonstrated the most favorable outcomes in the management of sarcopenia [101,102]. Practical integration may be facilitated by embedding muscle mass assessments into routine nutritional monitoring, offering flexible, home-based or low-intensity activity programs, and fostering interdisciplinary collaboration between oncologists, dietitians, and physiotherapists [103,104]. Early identification and individualized intervention can help prevent long-term functional impairments in this vulnerable population.

It may seem surprising that sarcopenia is observed even in pediatric cancer survivors, years after the completion of therapeutic interventions; yet, various studies attempt to explain this phenomenon. In the study by Nakayama et al. (2021), it was reported that patients who underwent hematopoietic stem cell transplantation for pediatric lymphoma or leukemia developed sarcopenia due to structural and metabolic damage to the muscles [39]. Furthermore, Kallenbach et al. (2022) investigated the long-term inflammatory and fibrotic alterations caused by radiotherapy, which resulted in the development of sarcopenia for a considerable period following recovery [105].

Sarcopenia is associated with long-term complications, and its timely diagnosis contributes to their prevention. In the study by Malhotra et al. (2021) [42], 14% of the patients had sarcopenic obesity—a precursor to cardiometabolic syndrome. The diagnosis of this combined condition enables the development of strategies to prevent the development of the syndrome [42]. Importantly, sarcopenic obesity itself is heterogeneous. Alalwan (2020) [106] distinguishes between visceral and subcutaneous sarcopenic obesity phenotypes, each with different clinical implications. Visceral sarcopenic obesity, which is characterized by excess fat around internal organs, has been linked to higher cardiometabolic risk, whereas subcutaneous sarcopenic obesity, with fat predominantly beneath the skin, may have different prognostic implications.

Pranikoff et al. (2022) [107], in their study, observed a higher frequency of frailty in childhood cancer survivors. In addition to the physical limitations and challenges associated with frailty, these survivors also reported poorer emotional and social health compared to the general population [107].

Multicenter prospective studies are required to implement standardized diagnostic criteria for sarcopenia in pediatric oncology, incorporating not only muscle mass but also functionality and physical activity [8]. The use of tools such as DEXA and pQCT, as well as the early introduction of therapeutic exercise during treatment, has been shown to be safe and effective [108].

It is therefore evident that sarcopenia is a common pathological condition, which may result either from the malignancy itself—thus being diagnosable before the initiation of treatment—or from the therapeutic interventions, when it is diagnosed in childhood cancer survivors. Its diagnosis is important, as it serves as a prognostic factor, contributing to the implementation of immediate measures aimed at halting its progression, and, finally, the comorbidities it causes can potentially be prevented.

It is obvious that there is substantial diagnostic heterogeneity among the studies summarized in Table 2, which likely contributes to variability in the reported prevalence and prognostic associations. Several of the included investigations employed computed tomography (CT)-based measurements (such as total psoas muscle area—tPMA—and skeletal muscle index—SMI). tPMA has been proposed and validated as a practical, age-adjusted CT marker in pediatric cohorts and has been repeatedly associated with clinical outcomes (Ritz, in neuroblastoma/hepatoblastoma cohorts). SMI, which is likewise CT-based and usually normalized to body size, demonstrated prognostic value in studies of neuroblastic tumors and sarcomas (Kudo, Buğdaycı), but the thresholds and vertebral levels applied were not consistent across reports. Other studies relied on clinically accessible techniques such as bioelectrical impedance analysis (BIA) (Romano), which is convenient due to its ease of use and suitability for repeated measurements, but is less accurate than imaging-based methods and can be influenced by hydration status and acute illness. Dual-energy X-ray absorptiometry (DXA) and peripheral quantitative computed tomography (pQCT) were used in some cohorts to assess lean mass and bone parameters; these offer low radiation exposure and the ability to evaluate whole-body or site-specific lean mass but may fail to detect regional muscle changes that are better captured by localized CT measurements. All of these methodological differences (technique, anatomical level, normalization method, and reference cut-offs) limit direct comparability between studies and may explain some conflicting findings; therefore, the development of pediatric-specific criteria and guidelines for measurement methods, anatomical landmarks, and age- and sex-adjusted thresholds is necessary to improve comparability and strengthen the robustness of future evidence.

Our results should be interpreted with some caution given the limitations of our design. First, the articles included in this review were retrieved from MEDLINE and Scopus; therefore, studies indexed exclusively in other databases are not represented. Second, sarcopenia was not diagnosed using a consistent method across the included studies, which should be acknowledged and considered when interpreting the main results.

## 5. Conclusions

Based on the total findings of the studies included in this review, sarcopenia is a frequent and clinically important condition in pediatric cancer patients. Sarcopenia appears both after treatment and even before its initiation, thereby increasing the risk of relapse and unfavorable outcomes. Reduced physical activity, insufficient caloric intake, and the effects of medications mainly contribute to its development. It is essential to prevent the development of sarcopenia to achieve and improve the survival of childhood cancer patients. Nevertheless, the results of this review should be interpreted with caution. The studies included show significant heterogeneity in diagnostic methods, cutoff values, and patient populations, which limits comparability and prevents firm conclusions about sarcopenia prevalence and optimal assessment tools. Future multicenter prospective studies applying standardized diagnostic criteria are essential to accurately determine prevalence and guide effective interventions in this vulnerable population.

## Figures and Tables

**Table 1:** PICO table outlining Population (P), Intervention (I), Comparator (C), and Outcome (O).

PICO	
Population (P)	Children and adolescents diagnosed with cancer (all types), during or after treatment, that appeared sarcopenia
Intervention (I)	Factors associated with the development of sarcopenia: chemotherapy, radiotherapy, corticosteroid use, malnutrition, reduced physical activity
Comparator (C)	Pediatric cancer patients without sarcopenia, or healthy age-matched controls.
Outcome (O)	Positive association between pediatric cancer/oncology and sarcopenia.

**Table 2:** Table summarizing the articles retrieved based on PRISMA flow diagram.

#	Author	Date of Publication	Type of Study	Type of Cancer	Population	Results
1	Ritz A et al. [49]	May 2021	Research Article	Hepatoblastoma	67 children from the Dr. von Hauner Children’s hospital with an average age of 2.15 years	Association between hepatoblastoma and sarcopenia, which leads to relapse. The measurement of tPMA was proposed for the assessment of sarcopenia. Sarcopenic children were more numerous than non-sarcopenic ones and were associated with a higher likelihood of relapse.
2	Romano A et al. [50]	Nov 2024	Research Article	Brain Tumor	14 male childhood brain tumor survivors, above 12 years	Association of pediatric brain cancer with sarcopenia and metabolic syndrome (MetS). The use of BIA for assessing body composition and fat content.
3	Das G et al. [44]	Nov 2024	Research Article	Acute Lymphoblastic Leukemia (ALL)	65 survivors of ALL between 7 and 18 years old.	Sarcopenic obesity that develops in survivors is an indicator of metabolic disease, and early exposure to anticancer treatment—which affects muscle health—also contributes to its development.
4	Kudo W et al. [51]	Aug 2024	Research Article	Neuroblastic Tumor	35 patients with an average age of 2.5 years	SMI served as a prognostic indicator and decreased during treatment, similar to HT and BW, which reflect growth. BMI did not follow the same pattern as the other parameters.
5	Marmol-Perez A et al. [52]	Aug 2024	Research Article	General Pediatric Cancer	116 young pediatric cancer survivors with an average age of 12.1 years	Approximately one third of the survivors developed sarcopenia, and these individuals had a higher likelihood of being assessed with low bone mineral density (BMD) values.
6	Suzuki D et al. [53]	Jan 2023	Research Article	Hematologic malignancies	65 patients, with a mean age of 11.3 years for males (40 individuals) and 11.7 years for females (25 individuals).	Patients who experienced loss of muscle mass (sarcopenia) prior to hematopoietic cell transplantation (HCT) were associated with poor overall survival. These patients had previously undergone chemotherapy.
7	Romano A et al. [54]	Jan 2022	Pilot retrospective study	Bone and soft tissue sarcomas (Ewing sarcoma, rhabdomyosarcoma, desmoplastic tumor)	22 pediatric patients age between 1 and 16 years	In many cases, sarcopenia appears at diagnosis. Evidence of a decrease in tPMA (sarcopenia measurement) 12 months after treatment. No association was found with prognosis.
8	Ritz A et al. [55]	Aug 2021	Retrospective analysis	Neuroblastoma	101 children between 1 and 15 years of age, who underwent a workup for NB	The majority of patients showed reduced tPMA before surgery. Sarcopenia is associated with reduced prognosis.
9	Guo M et al. [56]	Aug 2021	Prospective Study	High Risk-Neuroblastoma	20 survivors of HR-NBL 6–16 years and 20 healthy controls	Survivors of high-risk neuroblastoma treated with cis-retinoic acid exhibit sarcopenia and diminished skeletal growth years after treatment. Sarcopenia and sarcopenic obesity are indicators of poor prognosis.
10	McCastlain K et al. [57]	Apr 2021	Research Article	General Pediatric Cancer	1762 Survivors aged 18 years and older at follow-up, and 10 or more years from primary diagnosis	The decrease in mtDNAcn after cancer treatment is indicative of sarcopenia. Risk factors for sarcopenia include female gender, tumor type, age at diagnosis, exposure to cranial radiation and alkylating agents, physical inactivity, Asian ancestry, and the presence of the T allele at the genetic locus rs9991501 (in the HSD17B11 gene).
11	Van Atteveld JE et al. [58]	Apr 2023	Research Article	General Pediatric Cancer	3996 adult survivors aged between 18 and 45 years old	The occurrence of frailty, pre-frailty, and sarcopenia has been observed in survivors, even in the third decade of life following treatment for childhood cancer.
12	Buğdaycı O et al. [59]	2023	Research Article	Ewing Sarcoma and Osteosarcoma	60 patients aged between 16 months and 18 years.	There is no clear association between overall survival, with or without events, and sarcopenia, although a higher skeletal muscle index (SMI) was associated with increased survival in survivors.
13	Omori A et al. [60]	Dec 2022	Retrospective cohort study	Malignant Solid Tumors	progression-free survival group (PFS group) (n = 21), relapse/death group (R/D group) (n = 7). Control 185	Before the start of treatment, patients with tumors did not show sarcopenia. Increased muscle mass after the end of treatment is an indicator of good prognosis after treatment.

Data Synthesis: Due to expected heterogeneity in different study designs, a qualitative synthesis of the above-mentioned variables (without statistical synthesis) was chosen a priori. We grouped eligible studies into categories for summarization and better result interpretation.

**Table 3:** Assessment of methodological quality of cohort studies, according to the adapted Newcastle–Ottawa Scale (NOS).

References	Selection				Comparability		Outcome		Total Quality Score
	Representatives of Exposed Cohort	Sample size	Assessment of Outcome	Non-Respondents	Adjust for the Most Important Risk Factors	Adjust for Other Risk Factors	Assessment of Outcome	Statistical Test	
Ritz A et al. [49]	1	1	1	1	1	0	1	1	7
Romano A et al. [50]	1	0	1	1	0	0	1	1	5
Das G et al. [44]	1	1	1	1	1	0	1	1	7
Kudo W et al. [51]	1	1	1	1	1	0	1	1	7
Marmol-Perez A et al. [52]	1	1	1	1	1	1	1	1	8
Suzuki D et al. [53]	1	1	1	1	1	0	1	1	7
Romano A et al. [54]	1	1	1	1	1	0	1	1	7
Ritz A et al. [55]	1	1	1	1	1	0	1	1	7
Guo M et al. [56]	1	1	1	1	1	0	1	1	7
McCastlain K et al. [57]	1	1	1	1	1	1	1	1	8
Van Atteveld JE et al. [58]	1	1	1	0	1	1	1	1	7
Buğdaycı O et al. [59]	1	1	1	0	1	0	1	1	6
Omori A et al. [60]	1	1	1	0	1	0	1	1	6

Methodological quality classification based on total score: <5: low quality; 5–7: moderate quality; >7 high quality.

## Supplementary Materials

The following supporting information can be downloaded at: https://www.mdpi.com/article/10.3390/cancers17193188/s1, Figure S1: PRISMA checklist.
